# Factors restraining the population growth of *Varroa destructor* in Ethiopian honey bees (*Apis mellifera simensis*)

**DOI:** 10.1371/journal.pone.0223236

**Published:** 2019-09-26

**Authors:** Haftom Gebremedhn, Bezabeh Amssalu, Lina De Smet, Dirk C. de Graaf

**Affiliations:** 1 Laboratory of Molecular Entomology and Bee Pathology, Ghent University, Ghent, Belgium; 2 Tigray Agricultural Research Institute, Mekelle, Ethiopia; 3 Holeta Bee Research Center, Holeta, Ethiopia; CNRS, University Paul Sabatier, FRANCE

## Abstract

Worldwide, the ecto-parasitic mite *Varroa destructor* has been assigned as an important driver of honey bee (*Apis mellifera*) colony losses. Unlike the subspecies of European origin, the honey bees in some African countries such as Uganda and Ethiopia may not be as threatened or suffer less from mite-infestations. However, only little is known about the factors or traits that enable them to co-exist with the mite without beekeepers’ intervention. Hence, this study was designed to investigate these factors or traits that limit the *Varroa* mite population in Ethiopian honey bees (*Apis mellifera simensis*). The study was conducted in the primary honey producing region of Ethiopia, i.e. Tigray. Mite infestation levels were shown to be lower in traditional hives (when compared to framed hives) and when colonies were started up from swarm catching (when compared to colony splitting). However, the influence of the comb cell size on mite infestation was not observed. With respect to the bee biology, the hygienic behavior was shown to be high (pin-test: 92.2% removal in 24 hours) and was negatively correlated with phoretic mite counts (Pearson; r = -0.79; *P* < 0.01) and mite infestation levels in brood (Pearson; r = -0.46; *P* < 0.001). Efforts to estimate the *Varroa* mite reproductive capacity were seriously hampered by an extremely low brood infestation level. From the 133 founder mites found (in 6727 capped brood cells) only 18.80% were capable of producing a reproductive progeny. Failure to produce adult male progeny was unexpectedly high (79.70%). We have suggested a few adaptations to the test protocols allowing to estimate the protective traits of honey bee colonies under very low *Varroa* pressure. Apart from that, this study demonstrates that the honey bees from Ethiopia are suitable targets to further decipher the genetic predisposition of resistance against *V*. *destructor*. It is still unclear to what extent *simensis* differs from the more common *scutellata* subspecies.

## Introduction

The contribution of honey bees to agricultural production [1], food security [2, 3], nutrition, income in households [4] and ecosystem services [5] is significant. However, beekeepers are experiencing high colony losses, especially in developed countries [6–8] which are attributed to many interacting factors, including honey bee diseases [8–10], pests [8], pesticides [7, 11] and nutritional stress [6, 12]. Of these, the ecto-parasitic mite *Varroa destructor* has been assigned as one of the most important causal factors [6, 7] and for decades beekeepers from Europe and the USA mainly rely on treatment with acaricides to control mite infestation levels [13–15].

The presence of the *Varroa* mite has been confirmed in many African countries, including Ethiopia [16–21]. Unlike the subspecies of European origin, the local honey bee subspecies may not be as threatened or suffer less from mite-infestations in African countries such as Uganda, Ethiopia, Kenya and Nigeria and the impact of varroatosis in these populations is rather limited [4, 17, 22–24]. African bees can survive for extended periods without the use of mite control treatments or beekeepers’ intervention [25–27]. However, the factors or traits that enable honey bees in some African countries to co-exist with *V*. *destructor* have only recently been studied [27–29].

In the present study, we investigated explanatory factors related to 1) the apicultural management and 2) the honey bee biology (behavior and physiology), to try understanding the factors that limit the *Varroa* mite population in Ethiopian honey bees (*Apis mellifera simensis)*. We undertook this research to give the local beekeepers documented advice about their beekeeping management and to better understand the traits or factors that provide bee populations resilience against the *Varroa* mite. Beekeepers’ management techniques can influence (i.e. favorably or unfavorably) the burden of parasites and pathogens in a colony. But, beekeepers are often not aware of the harmful consequences of their interventions and it is therefore important to properly assess the risks of the management techniques in apiculture. A typical management factor that could contribute to the host-parasite equilibrium between the *Varroa* mite and the honey bee is the smaller comb cell size build by bees. A smaller comb cell size reduces the amount of space between the developing bee and the cell wall [30, 31] and shortens the honey bees’ developmental time in the capped brood [32], which consequently negatively influences the mites developmental success [33]. The beekeepers have control over this by offering empty frames without wax foundations as it will force the bees to build smaller brood cells. However, comb building without wax foundations has an energetic cost which eventually will reduce the honey yield. Thus, beekeepers must make choices in their business methods and weigh up the pros and cons. Other factors related to the beekeeping practice that were examined are the hive type (traditional *versus* framed hives) and the method of colony start-up (swarm catching *versus* colony splitting). Honey bees behavioral traits that might limit the growth of the *Varroa* mite population are high swarming [34], absconding tendencies [35], hygienic behavior (a social trait that consists of removing dead or/and infected pupae) [4, 27, 35–37] and grooming behavior [27, 35, 38]. Besides, a physiological trait that renders honey bees resistant to *Varroa* by reducing the mite reproductive success is also described [28, 29, 35, 39]. In this ‘suppressed mite reproduction’ (SMR) trait the *Varroa* mite fails to produce offspring, which has an important impact on the mite population dynamics [40]. The underlying mechanism is not entirely clear, but the trait is exclusively expressed by the late larval or pupal stage [35, 41] as the mite reproduction takes place in the sealed brood cell. Moreover, it seems to occur both in worker and drone brood [35, 41] and has been reported in bees left untreated in Europe and in *Apis mellifera scutellata* in Africa [28, 29, 39–41]. Our research group has recently unlocked the genetic predisposition of the SMR trait of honey bees of the Amsterdam Water Dunes (The Netherlands) by combining whole exome sequencing and elastic-net regression [41]. A molecular mechanism has been proposed in which the chemical communication between the honey bee larva and the *Varroa* mite is disturbed, and as a consequence hereof the onset of mite oogenesis failed. We believe that the overall mechanism is universal, whereas the genes involved may differ between the honey bee strains/races/subspecies. We therefore are seeking for unexplored honey bee populations that became resistant against *Varroa* infestations by natural selection. In this context, the Ethiopian honey bees are very interesting targets.

## Materials and methods

Before commencing the study, the research proposal endorsement was obtained from the Livestock research director review committee of the Tigray Agricultural Research Institute (No. 13839/ET-27/19). Verbal informed content was obtained from all participants prior to each respondent being interviewed and they were advised that they were free to participate or not participate in the interview.

### Study area

**Fig 1:**
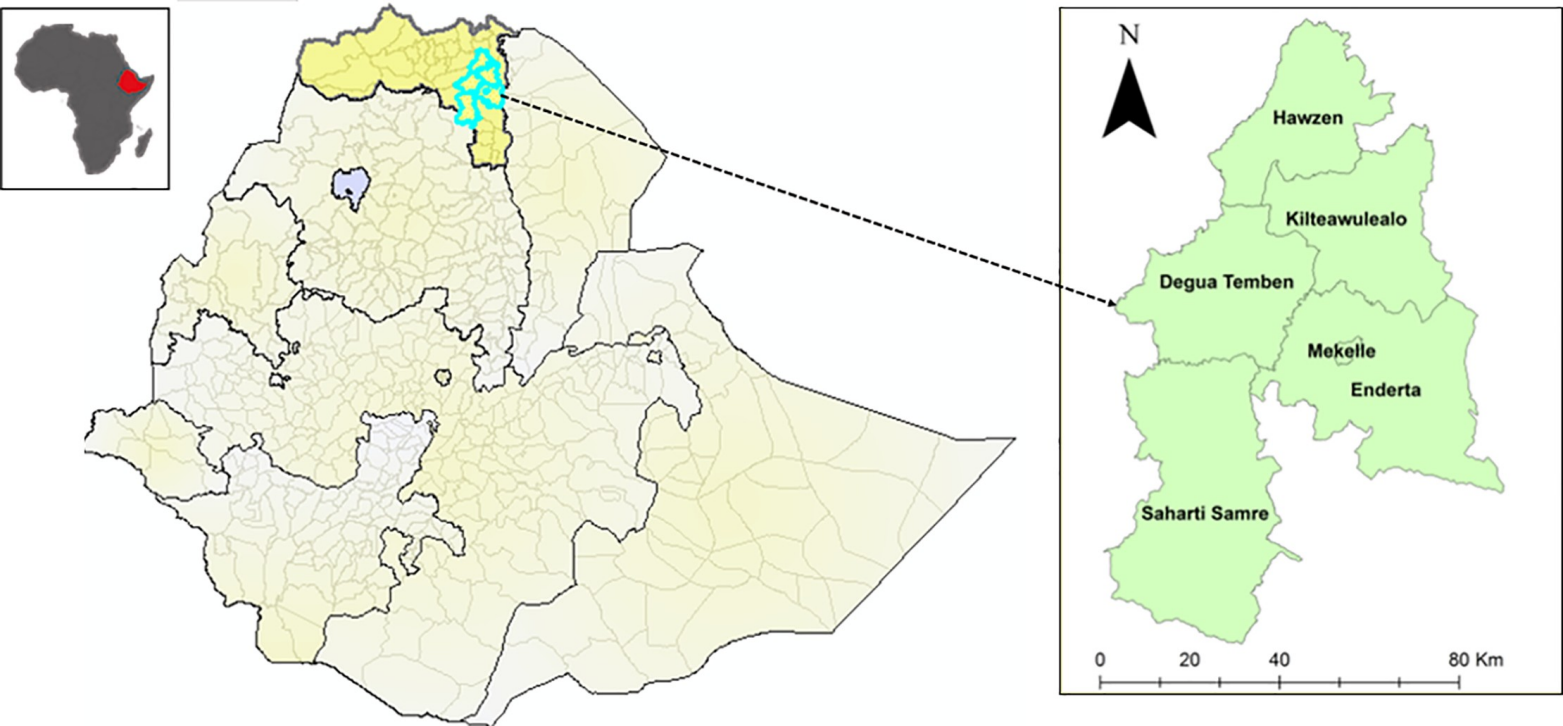
Map of Ethiopia in the horn of East Africa (see left window). In the right window the selected districts in the Tigray National, Regional State of Ethiopia are shown. Parts of this figure are downloaded from Shutterstock with an Enhanced Licence Subscription.

The study was conducted from May 2017 to February 2018 in the Federal Democratic Republic of Ethiopia, more in particular in the Tigray region (Fig 1). The country is located in the horn of East Africa and characterized by diverse agro-climatic conditions [42–45]. In addition to the numerous feral honey bee colonies, there are about 5.92 million colonies housed in traditional (96.46%), transitional (1.24%) and movable framed (3.34%) hives [46]. The new classification regarding the Ethiopian subspecies indicated that *A*. *mellifera simensis* is the only honey bee subspecies found in the nation [47].

### Sample collection

The influence of beekeeping management practices such as hive type, cell size, method of colony start-up and absconding tendency on limiting the growth of *Varroa* were studied in the districts Mekelle, Hawzen, Kilte Awlaelo, Sahrti Samre, Degua Temben and Enderta (Fig 1). These districts were selected on the basis of access to transport, the existence of traditional and/or movable framed hives and the prevalence of *Varroa* mites in these areas [16, 48]. Samples (adult bee and/or capped brood) were collected from apiary sites of beekeepers, farmers training centers (FTC), a research center, university and agricultural college (S1 Table). These samples were collected from May to July 2017 after getting permission (i.e. to collect samples) from the owner of the site. Adult bees and capped brood were collected to determine the mite infestation levels. The samples were stored at 4°C until further examination at the Tigray Agricultural Research Institute (TARI), in Mekelle, Ethiopia.

### Experimental colony establishment

To evaluate the hygienic behavior, grooming behavior and *Varroa* mite reproduction (see below), 24 honey bee colonies (*A*. *m*. *simensis*) in movable framed hives were purchased from the local beekeepers of Kilte Awlaelo, Dogua Temben and Mekelle districts, and transported to the apiary site of the Tigray Agricultural Research Institute, Mekelle (after getting confirmation from the owners; S1A Table). These districts were selected based on access to transport and the willingness of the beekeepers to sell whole honey bee colonies in movable framed hives. Once the colonies were properly established at the apiary site of the Tigray Agricultural Research Institute, Mekelle (June to July 2017) the observations were started. The evaluation was performed in both the active (August to September 2017) and dry (February 2018) season.

### Quantifying the *Varroa* infestation level

Measurements of the *Varroa*-infestation level and the behavioral and physiological traits were mainly in accordance to the test protocols of the Flemish breeding program [49], a procedure that is in line with that of other leading selection programs such as the German Beebreed program (http://www2.hu-berlin.de/beebreed/ZWS/) and European Commission-funded SMARTBEES program (http://www.smartbees-fp7.eu/). Prior to the experiments in Ethiopia, the involved researcher obtained practical training at the Ghent University apiary.

#### Phoretic mite counts

Adult bees were shaken of a brood comb and collected by filling one 250 ml container (which corresponds to a sample size of 257 ± 2.91 adult bees) per colony. The phoretic mites were dislodged from adult bees by washing in a water-detergent solution with vigorous shaking for 4–5 min [50]. Bees were separated from the mites by sieving (mesh size of approximately 2–3 mm). Bees and mites were counted and the infestation levels are expressed as the number of mites per 100 adult bees.

#### Brood examination

The *Varroa* infestation level of brood was determined by opening randomly 260 sealed brood cells. This number was set in order to estimate the brood *Varroa* infestation level on an equally large number of specimens as we did for the phorectic mite counts (257 ± 2.91 adult bees per colony). Infestation level was given as the number of mites per 100 opened brood cells (%).

### Beekeeping management

#### Hive characteristics

To study the influence of hive type on the *Varroa* infestation level, samples at the remote apiaries (S1B Table) were taken. In total 66 traditional hives and 33 framed hives were inspected and adult bees were sampled to determine the *Varroa* infestation level (May to June 2017). Here, the level of phoretic mites was used to estimate the *Varroa* load.

#### Influence of cell size

In order to study the influence of the cell size on the *Varroa* infestation level, 30 honey bee colonies from Hawzen, Kilte Awlaelo and Mekelle districts with framed hives which were completely disconnected from the experiment studying the influence of the hive type were used (S1C Table). *De novo* comb building was stimulated by introducing both an empty frame and a frame with wax foundation (with European embossed pattern) in the middle of the brood chamber (i.e. at the same time in the same colony) of the 30 colonies (May to July 2017), knowing that this would influence the resulting brood cell size. This experiment was performed in periods with a high availability of nectar which is the energy source for wax secretion. Because the construction and oviposition/brood care was not always successful, combined measurements of cell size (see above) and *Varroa* infestation level in capped brood could only be performed on 34 combs (i.e. 17 from frames with beeswax foundation and 17 from frames without beeswax foundation).

The brood combs were photographed in order to determine the brood cell size (hexagonal size and the maximum height) using the ImageJ software package [51]. To determine the cell size, the pixel values of each image were converted to cm by quantifying a cm scale with a known distance (ruler) photographed at the same magnification [52].

#### Method of colony start-up

The effect of colony start-up (swarm catching *versus* colony splitting) on the level of *Varroa* mite infestation of adult bees and in brood cells was studied. Information related to the method of colony start-up was determined by interviewing the responsible beekeeper or his/her technician who were free to participate (interview) (S1D Table). Pairs of colonies coming from the same apiary (i.e. established through splitting and swarm catching) were selected. Colonies should fulfill the following conditions: well established, similar in strength (i.e. population and brood level) and established in the same season but with a different start-up method. Sixteen such pairs were found distributed over six apiary sites of the Mekelle district (S1D Table).

### Honey bee biology

#### Absconding rate

The absconding rate of the local bees was determined based on the observations made on 10 apiary sites following 73 honey bee colonies in framed hives (S1E Table). The apiaries were visited for the first time between May and June 2017 to collect adult bees to determine the *Varroa* infestation level (pre-absconding). In February 2018 the same hives (i.e. marked at the first visit) were inspected for the presence of bees. An empty hive was interpreted as an absconding colony. The absconding rate was then correlated with the pre-absconding *Varroa* mite infestation level.

#### Hygienic behaviour

The hygienic behavior was determined by the pin-test [53] in the purchased 24 honey bee colonies (see above) during the active season. Approximately 100 pupae on a 5 by 5 cm area were deliberately damaged/killed by puncturing a needle through the cell capping. The frame was then photographed and reintroduced into the hive. The same patch of brood comb was photographed again 12 and 24 hours later and examined with the ImageJ software package in order to determine the number of brood cells that were emptied in the meantime. The hygienic behavior was expressed as the proportion of the damaged/killed pupae that were emptied over a 12 or 24-hour interval over the total number of damaged cells [54]. Measurements were repeated three times with a three-day interval. Due to limited brood availability, we could not perform measurements of hygienic behavior in the dry season.

#### Grooming behaviour

The grooming behavior (fallen and damaged mites) was measured in both the active (August to September 2017) and dry season (January 2018) from 24 and 17 honey bee colonies, respectively. Seven colonies were lost due to absconding. The hives were equipped with a bottom board that contained a wire sheet with 4 mm wide square holes, on which a white sheet of paper coated with Vaseline was placed [38]. Every two days the sheets were collected and immediately replaced with a new one. This was repeated three times. To avoid living mites to escape, the fallen debris was placed directly in a freezer. All the hives were placed on a stand with ant protection (i.e. ash and oil applied on stands) to avoid interference by entering ants. All the fallen mites were examined microscopically for any deformity at 40x magnification [27] and the kind of damage was classified according to previously described criteria [27, 55]. The degree of grooming was given as a percentage of damaged mites over the total number of fallen mites [27].

#### *Varroa* mite reproduction

The reproductive ability of the mother *Varroa* mite was determined on sealed worker brood. The examined pupae should have reached at least the dark grey headed stage [50, 56]. According to the protocol, only one sealed brood frame with approximately 200 brood cells should be sufficient to find 30 mite infested pupae of the desired age. However, during the execution of the experiment in Ethiopia the infestation was so low that more cells had to be opened. Eventually, an average of 280 ± 10.2 brood cells were opened per colony and only cells invaded by a single mother mite were considered. The adult female daughter was distinguished from their mother mite by their lighter pigmentation as previously described [56, 57]. *Varroa* mite fertility was determined by counting the number of mother mites laying at least one egg [50]. The number of mother mites producing viable female offspring was calculated by counting the number of mother mites that contain at least one adult male and at least one daughter mite [50]. For this purpose, the presence or absence of an adult male and female offspring per mother mite was recorded. Due to limited brood availability during the dry season, *Varroa* mite reproduction was measured only in the active season.

### Statistical analysis

In the present study, we performed parametric and non-parametric statistical analysis. Mann-Whitney U test was performed when group values violated the normal distribution. The influence of hive type (framed *versus* the traditional hive), method of colony start-up (splitting *versus* swarming), *de novo* building activity (frames with wax foundation *versus* frames without wax foundation) and absconding tendency (absconded *versus* not-absconded) on *Varroa*-infestation levels, and the grooming behavior (fallen mites and damaged mites) in relation to season (i.e. the active and dry season) was performed using the Mann-Whitney U test. The analysis was performed by considering each parameter as a factor. The independent t-test was used to compare *Varroa* infestation levels between the active and the dry season after transforming the values using log+1 (i.e. to normalize the data). To associate the hygienic and grooming behavior with *Varroa* infestation levels, the Pearson correlation and a linear regression model were used. For the multiple tests we performed a Bonferroni correction in order to correct for type I error. The statistical analyses were performed using SPSS version 20. Graphs were developed using Excel (2016).

## Results

### Beekeeping management

Our study demonstrates that different management practices have an influence on the *Varroa* infestation levels (Table 1). Honey bees kept in traditional hives have relatively lower levels of phoretic *Varroa* mites (1.14 ± 0.21 *versus* 2.40 ± 0.28 in framed hives; *P* = 0.003; Table 1). In order to determine the effect of brood comb cell size on the *Varroa* infestation level, frames with or without wax foundations were inserted in the colonies. *De novo* frame building resulted in brood cells of different sizes; smaller cell sizes were observed in frames without wax foundation sheet (Table 1). However, no differences in *Varroa* infestation level could be observed between combs built with or without beeswax foundation (1.20 ± 0.35 *versus* 0.92 ± 0.19; *P* = 0.876; Table 1). Further, colonies started up from swarms had a lower number of phoretic *Varroa* mites compared to those started up from colony splitting (0.56 ± 0.12 *versus* 1.87 ± 0.56; *P* < 0.001; Table 1).

**Table 1:** Influence of different factors on *Varroa*-infestation level.

Factor	Variable	Category	N	Mean ± SE	Mean rank	Statistics			
Hive type	*Varroa*_Ad	Traditional hive	33	1.14 ± 0.21	38.03	U = 694.0	Z = -237	α’ = 0.0125	*P* = 0.003*
		Framed hive	66	2.40 ± 0.28	55.98				
Method of colony starts up	*Varroa*_Ad	Splitting	16	1.87 ± 0.56	22.88	U = 26.0	Z = -3.888	α’ = 0.0125	*P* < 0.001*
		Swarming	16	0.56 ± 0.12	10.13				
	*Varroa*_Br	Splitting	16	1.84 ± 0.32	20.56	U = 63.00	Z = -2.254	α’ = 0.0167	*P* = 0.025^ns^
		Swarming	16	0.41 ± 0.14	12.44				
*De novo* building activity	*Varroa*_Br	With foundation	17	1.20 ± 0.35	17.76	U = 140.0	Z = -0.156	α’ = 0.0167	*P* = 0.876
		Without foundation	17	0.92 ± 0.19	17.24				
	Hexagonal size	With foundation	27	5.34 ± 0.04 mm		t = -3.304	Df = 45		*P* = 0.002*
		Without foundation	20	5.12 ± 0.05 mm					
	Maximum height	With foundation	27	5.06 ± 0.06 mm		t = -3.939	Df = 45		*P* < 0.001*
		Without foundation	20	4.69 ± 0.06 mm					
Season	*Varroa*_Ad	Active season	24	4.02 ± 0.47§		t = 2.450	Df = 39	α’ = 0.0125	*P* = 0.019^ns^
		Dry season	17	2.69 ± 0.24§					
	*Varroa*_Br	Active season	24	3.94 ± 0.85§		t = 2.332	Df = 27.315	α’ = 0.0167	*P* = 0.027^ns^
		Dry season	17	1.84 ± 0.11§					
	Mites fallen	Active season	24	23.90 ± 3.07	66.16	U = 1423.5	Z = -1.90		*P* = 0.057
		Dry season	16	14.50 ± 2.16	53.91				
	Damaged mites	Active season	24	8.30 ± 1.12	63.51	U = 1609.5	Z = -0.925		*P* = 0.355
		Dry season	16	6.10 ± 0.88	57.56				
Absconding tendency	*Varroa*_Ad	Absconded	33	2.05 ± 0.42	39.32	U = 736.5	Z = -0.534	α’ = 0.0125	*P* = 0.593
		Not absconded	48	2.17 ± 0.28	42.16				

U = Mann-Whitney U test; Z = Z-score; α’ = Bonferroni corrected α’; *P* = probability value; Df = Degree of freedom; N = Number of colonies per each category; *Varroa*_Ad = *Varroa*-infestation level on adult bees (phoretic mites) (%); *Varroa*_Br = *Varroa*-infestation level in brood cells (either worker or drone brood) (%); in case of hygienic behavior the level of brood removal was recorded (%); grooming behavior was compared between the active and the dry season: first the number of fallen mites (per colony per 2 days) was given and subsequently the proportion of damaged mites (%).

§ = log^+1^ transformed values. The asterisks indicate a significant difference, while “ns” indicates non-significant difference after Bonferroni adjustment (α‘).

### Honey bee biology

With respect to the behavior of the Ethiopian honey bees, we found an absconding rate of 40.7%. The pre-absconding *Varroa* mite infestation level in colonies absconded and not absconded (i.e. after 8 to 9 months) was 1.92 ± 0.42 and 2.0 ± 0.28, respectively (Table 1) but the difference is not significant (*P* = 0.593).

**Fig 2:**
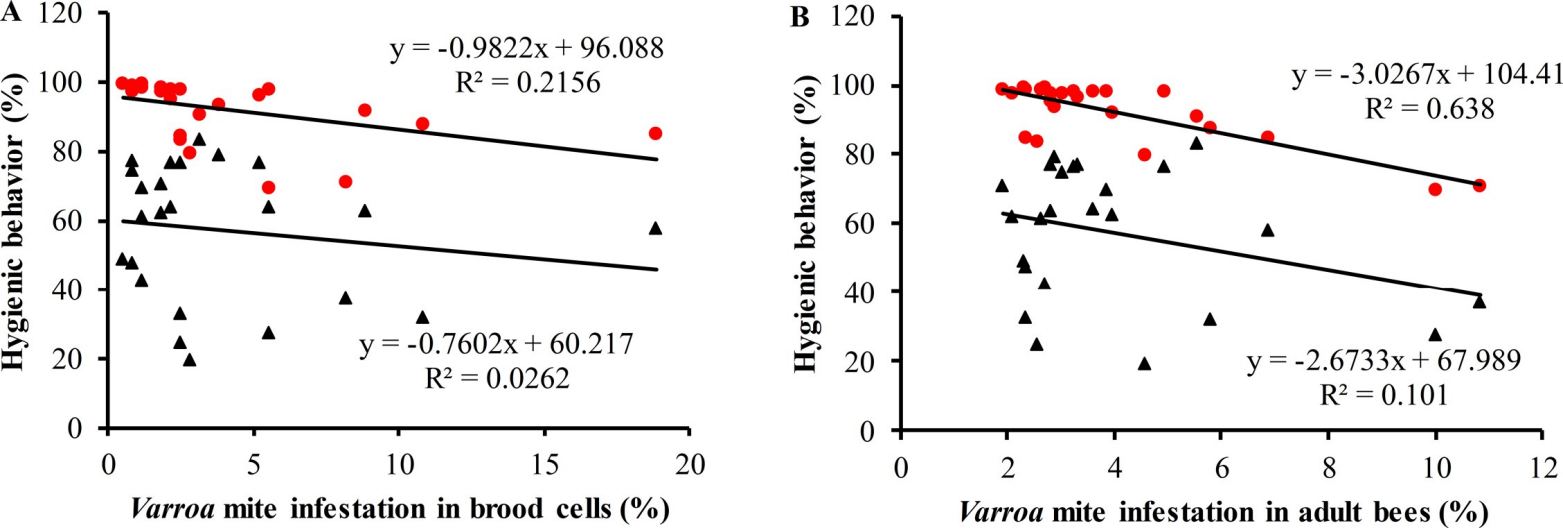
Correlation between the hygienic behavior (%) at 12 (black triangles) and 24 hr (red circles) and the *Varroa* mite infestation level (%) in brood cells (in A) and adult bees (in B).

After Bonferroni correction, the differences in *Varroa* infestation level between the active and the dry season were no more significant (Table 1). Related to the hygienic behavior, the local honey bees cleaned up on average 92.2 ± 1.81% and 57.20 ± 4.02% of the damaged/dead pupae over a 12 and 24-hours interval, respectively. The hygienic behavior (at 24 hr) was found to be negatively associated with both the *Varroa* infestation levels in brood cells (Pearson; r = -0.464; *P* < 0.001) and on adult bees (Pearson; r = -0.799; *P* < 0.01) (Fig 2). However, no such correlation was observed between *Varroa* mite infestation levels in brood cells (Pearson; r = -0.162; *P* = 0.45) and adult bees (Pearson; r = -0.318; *P* = 0.13) and the hygienic behavior after 12 hr (Fig 2). The result of the linear regression model also indicated that the variable, level of hygienic behavior explained 21.6% and 63.8% of the variance in the *Varroa* infestation level of brood cells (Fig 2A) and adult bees (Fig 2B), respectively.

**Fig 3:**
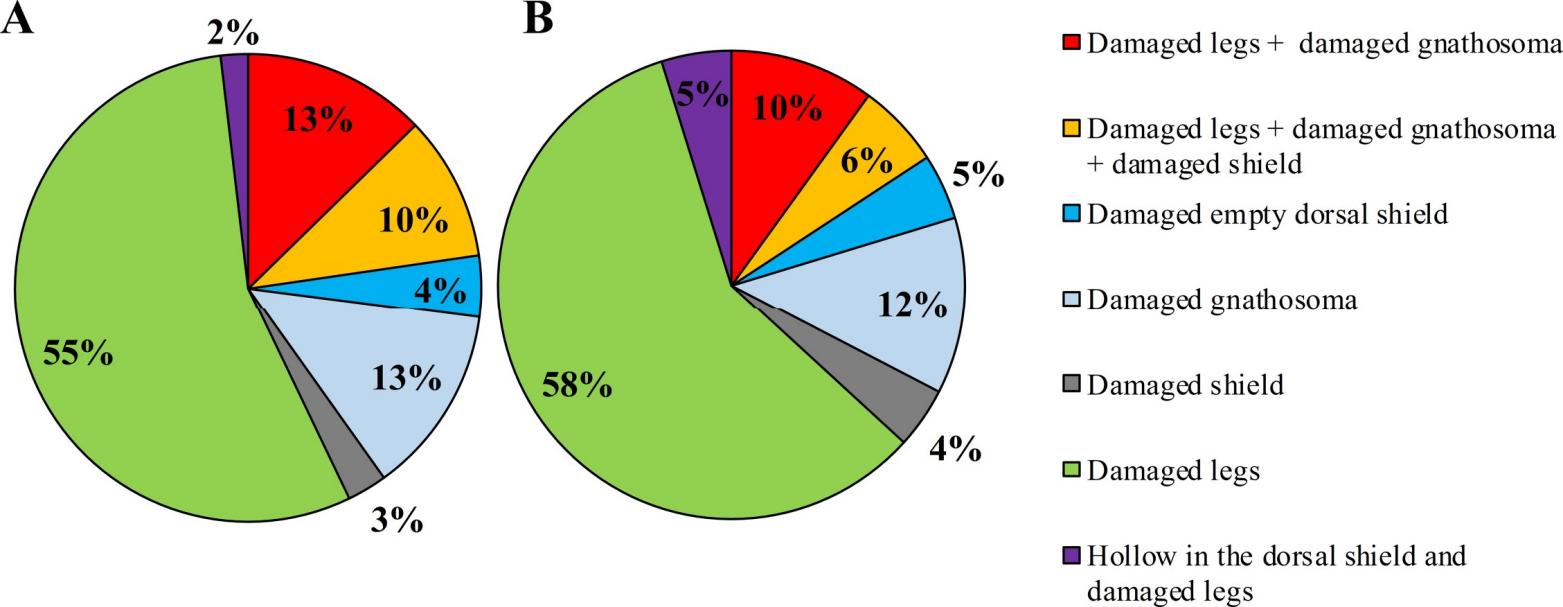
Grooming behavior. Distribution of the seven kinds of damages recorded of the mites collected at the bottom board during the active season (in A) and the dry season (in B).

A total of 1670 (69.6 ± 14.55 mites per colony) and 740 (43.5 ± 9.76 mites per colony) fallen mites originating from a total of 24 and 17 honey bee colonies were collected from the bottom board during the active and the dry season, respectively. However, significant differences were not observed between the active season and the dry season with regard to the number of mites fallen on the bottom board (U = 1423.5; Z = -1.90; *P* = 0.057; Table 1). In the present study, we considered the proportion of damaged mites as indicative of grooming behavior. Of the total number of mites fallen on the bottom board, 34.78% were damaged during the active season (N = 581) and 41.89% was damaged during the dry season (N = 310). There was no significant difference between the active and dry season related to the number of mites damaged (U = 1609.5; Z = -0.925; *P* = 0.355). In our study, seven kinds of damages were distinguished and damage of the legs was the most commonly recorded in both the active (55% of the damaged mites; Fig 3A) and dry season (58%; Fig 3B). During the active season, we did not find any significant association between the number of mites fallen on the bottom board and the *Varroa* infestation level in adult bees (Pearson; r = 0.371; *P* = 0.074) and the brood (Pearson; r = 0.0376; *P* = 0.071). Similarly, there was no significant correlation between the level of *Varroa* infestation in the adult bees (Pearson; r = 0.014; *P* = 0.923), the brood cells (Pearson; r = 0.184; *P* = 0.517) and the number of mites fallen on the bottom board during the dry season. The grooming behavior being the proportion of damaged mites, was not associated with the level of *Varroa* infestation in the adult bees (Pearson; r = 0.332; *P* = 0.19) and brood cells (Pearson; r = -0.021; *P* = 0.935) in the dry season and with the level of *Varroa* infestation in adult bee (Pearson; r = -0.193; *P* = 0.367) and brood cells (Pearson; r = 0.08; *P* = 0.71) during the active season.

In 6727 capped worker bee brood cells that were examined prior to the emergence of the bee, 133 founder mites were found (5.5 ± 0.58 mother mite per colony) (Table 2). Only 80 of them were accompanied by offspring (60.15%), the remaining 39.85% were considered as infertile. Altogether we collected 210 offspring; 108 adult female offspring (daughters; 51.43%), 28 adult male offspring (sons; 13.33%) and 74 immature offspring (35.24%). With 133 mother mites producing 108 adult daughters, the average female offspring produced per mother mite is only 0.81. Moreover, the number of mother mites that failed to produce a son was unexpectedly high: 106 (79.70%). The number of cells with females, but no males was also low: 38.3% (N = 51). Only the combination of adult male and female progeny can guarantee that eventually mated daughters (= reproductive progeny) emerge, and we found that only 25 out of 133 mother mites (18.80%) were capable of doing so.

**Table 2:** *Varroa destructor* counts in capped worker bee brood cells.

Category	N	Percentage
Total mother mites	133	100
Mother mites with offspring	80	60.15
Mother mites without offspring	53	39.85
Mother mites with adult male offspring	27	20.30
Mother mites without adult male offspring	106	79.70
Mother mites with adult female offspring	76	57.14
Mother mites without adult female offspring	57	42.86
Mother mites with adult male and female offspring	25	18.80
Total offspring	210	100
Adult male offspring	28	13.33
Adult female offspring	108	51.43
Immature offspring	74	35.24
Adult female offspring accompanied by adult male offspring	36	33.33
Adult female offspring not accompanied by adult male offspring	72	66.67

## Discussion

The present study aimed at determining the factors that influence the *Varroa* mite population and our results show that traditional hives have lower levels of phoretic mites, though the mechanism behind this remains unclear. Honey harvesting from the traditional hives occurs by removing the entire comb [58]. This management practice promotes *de novo* comb building and creates another internal environment in the hive, and one might speculate that this could influence the mite population dynamics. Earlier reports described already that old and new honey bee brood combs differ in *Varroa* infestation level and the hygienic behavior of the bees [59, 60]. We do not know whether this played a role here. However, the present study revealed a few interesting observations about *Varroa* load and beekeeping practices under Ethiopian circumstances, some of which might even be interconnected (hive type and method of colony start-up). The fact that we do not entirely understand their working mechanism and mutual relationship will encourage us to further explore this in the near further with a more target experimental design.

Our study could not support the evidence that cell size limits the growth of *Varroa* mite since there was no significant difference between the combs with beeswax foundation sheet (i.e. combs with bigger cell size) and without beeswax foundation sheet (i.e. comb with smaller cell size) related to mite infestation levels. This is consistent with some earlier reports [61, 62], but conflicts with others [63]. Consequently, we must look at the two mechanisms that try to explain the association between these two variables with a certain restraint, in particular 1) the reduced space between the pupae and the brood cell wall negatively affects the *Varroa* mite reproductive capacity [30] and 2) smaller brood cell sizes result in shortened honey bee developmental time [31, 32], and a reduced capping time potentially affects mite populations in honey bee colonies [64]. It seems that these two mechanisms do not explain entirely the relationship between brood cell size and *Varroa*-infestation level, whereby the association becomes circumstances-dependent.

African bees have a high absconding tendency [65]. The absconding rate of the examined Ethiopian bees (41.1%) is similar to those reported previously in Ugandan bees (38–45%) [17] and other African bees [66]. This trait negatively affects *Varroa* population dynamics as it creates a brood-free period. We found no correlation between the *Varroa* infestation level measured from May to June 2017, and their absconding behavior more than a half year later. The time point when the infestation level was determined may not be suitable to study this behavior. In the future, the infestation level should be determined on several time points to point out if there is a correlation between the infestation level and the absconding behavior. Moreover, the high absconding tendency of the local honey bees seems to be mainly associated with the low flowering intensity [67, 68], which we did examine neither.

Colonies established through swarming have a brood-free period, which may limit the development of pathogens and/or pests associated with brood. African bees are known to have a higher swarming tendency [65, 67] and also the local Ethiopian beekeepers make full use of this trait: 50–72% of them use swarm catching as a method for colony start-up [67, 69–71]. In the present study, we demonstrated that swarm catching should indeed be preferred over colony splitting as it results in lower mite infestation levels. This might be due to the presence of a brood free period when the colony is established through swarm catching. It is in line with earlier observations that *Varroa* infestation levels decrease with increasing swarming tendency of the bees [34]. Moreover, as colonies kept in traditional hives display a higher swarming tendency compared to those kept in framed hives [65], it may not surprise that we found the lowest *Varroa*-infestation levels in adult bees in traditional hives (1.14 ± 0.21) compared with the framed hive (2.4 ± 0.28).

A trait that receives much attention in the modern honey bee breeding, especially in the context of disease resistance, is the hygienic behavior. It is performed by bees between 15–20 days of age and consists of detecting, uncapping and removing infected brood [72]. Colonies that express this behavior are economically important to beekeepers as the trait was shown to limit the spread of bacterial (*Paenibacillus larvae*) and fungal (*Ascosphaera apis*) diseases [73]. With respect to brood infestation by *V*. *destructor*, is was found to limit the spread of the infection and to slow the reproductive potential of the mite [74, 75]. We used two time windows (12 and 24 hrs) in order to make measurements across a wide range of hygienic behavior possible: the shorter the time window, the stricter the criteria. In general, colonies that are capable of removing 90–95% of the pin-killed brood within 24 hr [76] and more than 95% after 48 hr are considered hygienic [77, 78]. In this study, the Ethiopian honey bees cleaned up on average 92.2 ± 1.81% of the damaged/dead pupae in a pin-test performed over a 24-hour interval, with more than 70% of the colonies reaching a removal rate above 90%. Another study in Southern Oromia, Ethiopia, obtained very similar results [79]. It seems that the hygienic behavior of Ethiopian bees is higher compared to that of other African bees in Kenya (65.5%) [4] and Egypt (72.5%) [54]. The negative association that we found between hygienic behavior (pin-test after 24 hr) and the mite infestation level in brood and on adult bees is in correspondence with the findings of Muli and colleagues on Kenyan bees [4] and reinforces earlier claims that hygienic behavior is one of the driving forces in the defence of African bees against pests and diseases [4, 27, 35]. We failed to see this beneficial effect when the pin-test was performed over a 12-hour interval, probably because the time window was too short. In Africanized honey bees, the hygienic behavior is also a trait that correlates with their resistance against the mite [36]. In our study, seven kinds of damages to the *Varroa* mites could be distinguished and it seems reasonable to believe that they are the result of grooming behavior as they perfectly match the recent classification of the damage caused by this behavior [27]. Like the work of Nganso and colleagues in *A*. *m*. *scutellata* in Kenya [27] our study could not find any evidence that grooming behavior contributes in any way to the defence of Ethiopian bees against the *Varroa* mite.

The most interesting observation in the present study is the capacity of the Ethiopian honey bees to suppress mite reproduction. With a mite reproductive success of 0.27 (viable female produced per mother mite or adult female offspring accompanied by adult male offspring (i.e. to mate with sister mites) [80]), Ethiopian bees surpass by far the values of European bees in England (1.01), and those of Africanized bees in Brazil (0.64) and Mexico (0.73) [80]. However, we acknowledge that our calculations are based on a limited set of data, due to the extremely low *Varroa* infestation level in the brood. The present study identified the inability to produce adult male offspring as a putative mechanism to suppress mite reproductive success and defence against the *Varroa* mite. Similar observations were recently done with *scutellata* bees in South Africa (0.3 ± 0.7) [28]. In our study, we did not differentiate between male or female immature mite stages, so we cannot exclude male mites that were produced, but did not reach adulthood as demonstrated elsewhere [81]. As the dark grey headed pupae is less than one day away from emergence, the remaining developmental time of the immature male mites is most probably too short to reach adulthood [82]. Normally, the last moult of the male mite is at the yellow thorax pupal stage of the honey bee, two days earlier [50]. Thus, when no adult male mites are found in the dark grey headed stage, it means that the male development is at least strongly delayed. Although several studies demonstrate that honey bees left untreated in Europe also develop suppressed mite reproduction [39, 41], the inability to produce adult male offspring has so far not been discovered outside Africa (except Africanized bees in Costa Rica; [81]).

This study also aimed at finding a bee population that was suitable to continue our search for the genetic predisposition of resistance against *V*. *destructor* infestations [41]. The honey bees in Ethiopia are eligible for this for several reasons: 1) the *Varroa* burden of these bees is particularly low and 2) both hygienic behavior and suppressed mite reproduction form the basis of their resistance against the *Varroa* mite. Further research is needed to fully understand the process and the present study demonstrates that we can no longer count on natural exposure to the mite for this. Indeed, when honey bee colonies are under very low *Varroa* pressure the test protocols should be adapted in order to raise the mite exposure artificially and at the same time the accuracy of the test results. Two such adaptations are suggested: 1) exposure to a so-called ‘mite shower' in which living *Varroa* mites are administered to the colony [83] and 2) an individual infection with phoretic mites that consists of opening the brood cell capping and inserting a *Varroa* mite artificially [84]. In the end, the honey bee populations with the lowest *Varroa* burden are the most interesting to crack the genetic origin of resistance against the mite.

## Supporting information

S1 Fig **Correlation between number of fallen mites or percentage of damaged mites and the *Varroa* mite infestation level (%) in brood cells (in A, C, E and G) and adult bees (in B, D, F, and H).** A-D corresponds to the dry season, whereas E-H corresponds to the active season.
(TIF)

S1 Table Locations, administrative information (tabia/kebele, district and zone) and GPS coordinates of the apiaries involved in this study. (PDF)

S2 Table Questionnaire for beekeepers. (PDF)
