# Supplementary material for: Factors restraining the population growth of Varroa destructor in Ethiopian honey bees (Apis mellifera simensis)
Source: PLoS One. 2019 Sep 26;14(9):e0223236. doi: 10.1371/journal.pone.0223236 (PMC6762127; doi:10.1371/journal.pone.0223236)
Supplement: S1 Table — (PDF) [file pone.0223236.s002.pdf]

Table S1. Locations, administrative information (tabia/kebele, district and zone) and GPS coordinates of the apiaries involved in this study

A. Apiaries where we purchased honey bee colonies to study hygienic behaviour, grooming behaviour and *Varroa* mite reproduction

Location/Site	Tabia/Kebele	District	Zone	Latitude	Longitude
Ilala	Adi welel	Mekelle	Mekelle	13°29'33"	39°27'56"
Liuelom Apiary site	Wukro Town	Kilte Awlaelo	Eastern	13°47'21"	39°35'47"
Ayninbirkekin	Ayninbirkekin	Degua Temben	South Eastern	13°39'00"	39°10'21"

B. Apiaries where we studied the influence of hive type on *Varroa*-infestation level

Location/Site	Hive code	Tabia/Kebele	District	Zone	Latitude	Longitude
Adi Kisanded FTC	Adi1-Adi4	Adi Kisanded	Kilte Awlaelo	Eastern	13°50’57.9"	39°37’52.9"
Ato Aklilu apiary site	Ak2-Ak3	May Kado	Hawzen	Eastern	14°0’44.2"	39°23’54.74"
Aynalem FTC	Ay1-Ay5	Aynalem	Kilte Awlaelo	Eastern	13°46'7.2"	39°32'52.6"
Ayninbirkekin	DT1-DT11	Ayninbirkekin	Degua Temben	South Eastern	13°40'37.5"	39°14'23.3"
W/ro birhan apiary site	Br1-Br3	Hawzen Town	Hawzen	Eastern	13°58’24"	39°25’31"
Felege Mayat	E1-E2	Felege Mayat	Enderta	South East	13°36'12.9"	39°29'37.5"
Gijet	Sa1-Sa2	Gijet	Samre	South East	13°17'59.1"	39°11'55.8"
Giyorgis church	Gi1-Gi2	Aynalem	Mekelle	Mekelle	13°27'09"	39°29'03"
Liuelom Apiary site	Lu1-Lu4	Ketema	Kilte Awlaelo	Eastern	13°47'21"	39°35'47"
Ilala apiary site of Mekelle Agricultural Research Center	M1-M10	Adi welel	Mekelle	Mekelle	13°31'29"	39°30'08"

Adikisanded apiary site of Mekelle Agricultural Research Center	W1-W10	Adi Kisanded	Kilte Awlaelo	Eastern	13°50'22.9"	39°37'23.3"
Megab FTC	Me1-Me4	Megab	Hawzen	Eastern	13°55’36.8"	39°23’29.5"
Mekelle University, Endayesus campus	Mu1-Mu4	Aynalem	Mekelle	Mekelle	13°29'05"	39°29'04"
Mekelle Agricultural Mechanization and Rural Energy Research Center	GT1-GT4	Aynalem	Mekelle	Mekelle	13°58'11.1"	39°28'8.4"
Selam chigin	SC1-SC3	Selam	Hawzen	Eastern	13°28'23"	39°28'54"
Selam FTC	SL1-SL4	Selam	Hawzen	Eastern	13°57'12.8"	39°27'54.02"
Siluh FTC	Si1-Si4	Suluhu	Hawzen	Eastern	13°59'18.7"	39°27'56"
Wukro Agricultural TVET College	Co1-Co10	Wukro Town	Kilte Awlaelo	Eastern	13°46'56.37"	39°36'14.8"
Wukro chigin tabia	Wc1 Wc9	Wukro Town	Kilte Awlaelo	Eastern	13°47'09"	39°35'34"

C. Apiaries where we studied the influence of cell size on *Varroa*-infestation level

Location/Site	Hive code	Tabia/Kebele	District	Zone	Latitude	Longitude
Ilala apiary site of Mekelle Agricultural Research Center	CS-1-5	Adi welel	Mekelle	Mekelle	13°31'29"	39°30'08"
Adikisanded apiary site of Mekelle Agricultural Research Center	CS-6-8	Adi kisanded	Kilte awlaelo	Eastern	13°50'22.9"	39°37'23.3"
Wukro Felsi tabia	CS-9-11	Wukro Town	Kilte Awlaelo	Eastern	13°47'09"	39°35'34"
Ato Aklilu apiary site	CS-12-15	May kado	Hawzen	Eastern	14°0'44.2"	39°23'54.7"
Megab FTC	CS-16-17	Megab	Hawzen	Eastern	14°55'40.3"	39°23'39.6"

D. Apiaries where we studied the influence of colony start-up on *Varroa*-infestation level

Location/Site	Hive code	Tabia/Kebele	District	Zone	Latitude	Longitude
Jone apiary site	Jsp1, JSw1, JSp2, JSw2	Ketema	Mekelle	Mekelle	13°29'01 "	39°29'59"
Mekelle Agricultural Research Center-Ilala	Isp1, ISw1, ISp2, ISw2 ,ISp3, ISw3, ISp4, ISw4	Adi welel	Mekelle	Mekelle	13°31'29"	39°30'08"
Mekelle University, Endayesus Campus	MSp1, MSw1, MSp2, MSw2, MSp3, MSw3, MSp4, MSw3	Aynalem	Mekelle	Mekelle	13°29'05"	39°29'04"
Mekelle Agricultural Mechanization and Rural Energy Research Center	GSp1, GSw1, GSp2, GSw2	Aynalem	Mekelle	Mekelle	13°28'23"	39°28'54"
Aynalem-Giwergis	ASp1, ASw1, ASp2, ASw2	Aynalem	Mekelle	Mekelle	13°27'19"	39°29'30"
Fkre Apiary site	ASp3, ASw3, ASp4, ASw4	Daero	Mekelle	Mekelle	13°31'18"	39°26'04"

E. Apiaries where we studied absconding tendency

Location/Site	Hive code	Tabia/Kebele	District	Zone	Latitude	Longitude
Selam FTC	S1-S4	Selam	Hawzen	Eastern	13°57'12.8"	39°27'54.02"
Aklil apiary site	A1-A2	May Kado	Hawzen	Eastern	14°0'44.2"	39°23'54.7"
Megab FTC	M1-M5	Megab	Hawzen	Eastern	13°55'36.8"	39°23'29.5"

Siluh FTC	Si1-Si4	Siluh	Hawzen	Eastern	13°59'18.7"	39°27'56"
Adikisanded apiary site of Mekelle Agricultural Research Center	A1-A14	Adikisan	Kilte awlaelo	Eastern	13°50'22.9"	39°37'23.3"
Aynalem FTC	Ay1-Ay7	Aynalem	Kilte awlaelo	Eastern	13°46'7.2"	39°32'52.6"
Ilala apiary site of MARC	I1-I11	Adiwelel	Mekelle	Mekelle	13°31'29"	39°30'08"
Mekelle Agricultural Mechanization and Rural Energy Research Center	G1-G2	Aynalem	Mekelle	Mekelle	13°28'23"	39°28'54"
Aynbirkekin FTC	D1-D9	Aynbirkekin	Dogua temebn	South-East	13°40'37.5"	39°14'23.3"
Felege Mayat	E1-E7	Felege Mayat	Enderta	South East	13°36'12.9"	39°29'37.5"
Gijet	Sa1-Sa9	Gijet	Samre	South-East	13°17'59.1"	39°11'55.8"
Mekelle University apiary site, Endayesus campus	Mu1-Mu8	Aynalem	Mekelle	Mekelle	13°29'05"	39°29'04"
