# Supplementary material for: Factors restraining the population growth of Varroa destructor in Ethiopian honey bees (Apis mellifera simensis)
Source: PLoS One. 2019 Sep 26;14(9):e0223236. doi: 10.1371/journal.pone.0223236 (PMC6762127; doi:10.1371/journal.pone.0223236)
Supplement: S2 Table — (PDF) [file pone.0223236.s003.pdf]

Table S2. Questionnaire for beekeepers (ንመራብሕቲ ንህቢ ዝተዳለወ መጠየቕ)

The aim of this questionnaire is to explore the influence of colony start up (splitting versus swarming) on the *Varroa* mite infestation levels (ዕላማ ናይዚ ፅንዓት ምምቃልን ተፈጥራኣዊ ምውላድን ዕስለ ንህቢን ኣብ ምርባሕ ቫርዋ ማይት ዘለዎ ፅልዋ ንምንፃር).

1. General (ሓፈሻዊ)

1.1. Date of the interview (ዕለት):__________________________

1.2. Name of the respondent/Beekeeper (ሽም):________________________________

1.3. Region (ክልል):_________________________________

1.4. Zone (ዞን):____________________________________

1.5. District (ወረዳ):_________________________________

1.6. Kebele (ጣብያ):________________________________

1.7. Village (ቁሸት):_________________________________

1.8. Location (GPS coordinate of the site) (ኣቀጣጫ): ______________________, ______________________

2. Beekeeping management (ማናጂመንት)

2.1. Do you keep bees in framed hive? (ዕስለ ንህቢ ኣብ ዘመናዊ ቆፎ ኣለውኹም ዶ?)

1=Yes (እወ); 2=No (የብለይን)

2.2. Do you use swarm catching as colony source? (ብተፈጥራኣዊ ምርባሕ ንህቢ ዕስለ ንህቢ ተዋለዱ ዶ?)

1=Yes (እወ); 2=No (ኣየዋልድን)

2.3. Do you use splitting to multiply your colonies? (ብምምቃል ዕስለ ንህቢ ተዋለዱ ዶ?)

1=Yes (እወ); 2=No (ኣየዋልድን)

2.4. Do you have colonies established through splitting in the last active season (July to September 2016)? (ኣብ ዝሓለፈ ዓመት ፤ ካብ ሓምለ - መስከረም 2008 ዓ/ም ብምምቃል ዝተዋለዱ ዕስለ ንህቢ ኣለውኹም ዶ?)

1=Yes (እወ); 2=No (የብለይን)

2.4.1. If yes, how many colonies did you split? (እወ እንተይሎም ፤ ክንደይ ዕስለ ንህቢ?)

____

2.5. Do you have colonies established through swarming in the last active season (July to September 2016)? (ኣብ ዝሓለፈ ዓመት ፤ ካብ ሓምለ - መስከረም 2008 ዓ/ም ብተፈጥራኣዊ መንገዲ ባዕሎም ተዋሊዶም ዝሓዘክምዎም ኣለውዶ?)

1=Yes (እወ); 2=No (የብለይን)

2.5.1. If yes, how many? (እወ እንተይሎም ፤ ክንደይ ዕስለ ንህቢ?) __________

2.5.2. If you establish colonies through swarm catching, did you give them brood combs for their establishment? (ባዕሎም ተዋሊዶም ንዝመፁ ሐሰኻ ትህብዎም ድኹም?)

1=Yes (እወ); 2=No (ኣይንህቦምን)
